# Expression of the Small Conductance Ca^2+^-Activated Potassium Channel Subtype 3 (SK3) in Rat Uterus after Stimulation with 17β-Estradiol

**DOI:** 10.1371/journal.pone.0087652

**Published:** 2014-02-05

**Authors:** Mette Rahbek, Sasan Nazemi, Lars Ødum, Saurabh Gupta, Steen Seier Poulsen, Anders Hay-Schmidt, Dan Arne Klaerke

**Affiliations:** 1 Department of Clinical Biochemistry, Roskilde Hospital, Roskilde, Denmark; 2 Department of Physiology, Biochemistry and Nutrition (IKVH), Faculty of Health and Medical Sciences, University of Copenhagen, Frederiksberg, Denmark; 3 Danish Headache Centre, Department of Neurology, Glostrup Research Institute, Glostrup Hospital, Faculty of Health and Medical Science, University of Copenhagen, Glostrup, Denmark; 4 Department of Biomedical Sciences, Faculty of Health and Medical Sciences, University of Copenhagen, Copenhagen N, Denmark; 5 Department of Neuroscience and Pharmacology, Faculty of Health and Medical Sciences, University of Copenhagen, Copenhagen N, Denmark; Medical Faculty, Otto-von-Guericke University Magdeburg, Medical Faculty, Germany

## Abstract

Preterm births accounts for roughly 9% of all births worldwide and can have detrimental or even lethal consequences for the infant. However to develop new treatment that will lower the rate of preterm births, more knowledge is required on the factors contributing to the contraction and relaxation of the myometrium. The small conductance Ca^2+^-activated potassium channel subtype 3 (SK3) has been identified in the myometrium of several species including humans, mice and rats, but with great inter species variation of the expression pattern and regulation. The aim of this study was to investigate the expression of SK3 in the uterus of rats stimulated with 17β-estradiol and progesterone in order to get an in depth understanding of the rat uterine SK3. Using immunohistochemistry SK3 was localized to the glandular and luminal endometrial lamina epitheliali. Furthermore, a weak signal was observed in the myometrium. Using Western blot the protein level of SK3 was found to increase in uteri from animals treated with 17β-estradiol, an effect that was not reflected at the mRNA level. The levels of mRNA for SK3 were significantly lower in the uterus of 17β-estradiol-treated animals than in the uterus of ovariectomized animals. We conclude that the SK channels are present in the endometrial epithelium, and possibly also in the myometrium of the rat uterus. Furthermore, the hormonal effect on SK3 caused by 17β-estradiol includes divergent regulation at mRNA and protein levels.

## Introduction

The smooth muscles of uterus, the myometrium, are relaxed throughout pregnancy and turns into a highly contractile muscle layer at term. Malfunctions of the relaxation and contraction pattern of the myometrium may lead to preterm labour which can have detrimental or even lethal consequences for the infant. Preterm birth have been reported to account for approximately 9% of all births worldwide [1], and 28% of all neonatal deaths are directly caused by preterm births [2]. Though many studies have already explored numerous aspects of the transition of the myometrium from relaxed to contractile state, a more detailed knowledge is needed in order to develop new tocolytics and to treat the condition.

Recently K^+^ channels and their role in the myometrium have been the subject of interest. By hyperpolarization of the cell following a depolarization, K^+^ channels are able to modulate the excitability and thereby the contractility of smooth muscle cells. Indeed several K^+^ channels have been identified in the uterus, where they contribute to the quiescence of the myometrium during pregnancy [3]–[6].

The small conductance Ca^2+^-activated K^+^ channel of subtype SK3 has been reported to be down-regulated both at mRNA level and protein level during normal pregnancy in the human myometrium. [7]–[9]. In genetically altered mice overexpressing SK3 protein a decrease in the phasic uterine contractions was observed and repression of SK3 with doxycycline resulted in increased contractile activity [10]. In addition, overexpression of SK3 protein has been shown to hinder normal parturition by diminishing contractility of the uterus [11]. In the rat myometrium no down-regulation was observed during pregnancy neither at mRNA level nor protein level [12]. Immunohistochemical studies have localized the SK3 channels to smooth muscle layer in both the rat and mouse uterus [10], [12]. A recent study on the human myometrium describe the localization of SK3 to the telocytes, an interstitial Cajal-like cell, but finds no staining of the muscle cells [13]. These discrepancies both between species and in localization indicate that a further characterization of SK3 in the myometrium is required.

Studies in mice have revealed that the amount of SK3 mRNA and protein in the uterus is down-regulated by estrogen [9]. Progesterone on the other hand has no effect on SK channel expression, as well as a range of other K^+^ channels, as shown by a study in the human myometrium [14].

In this study we have investigated the expression of SK3 in the rat uterus treated with 17β-estradiol or progesterone, in order to clarify if rat SK3 is hormone regulated as reported for mice and human. Furthermore, the localization of SK3 in the myometrium as well as in the endometrium, was investigated as species differences have been observed in the spatial (anatomical) distribution of the channels.

## Materials and Methods

### Ethics Statement

All the experimental protocols were approved by the Danish committee for experiments with animals (2009/561-1664). The use of hormone pellets in the animal was used to avoid any unnecessary stress for the animals.

### Animals and Tissue Collection

Female virgin Sprague Dawley rats, approximately 200 g (Harlan, Holland) were housed four and four with food pellets and water *ad libitum*. The rats were divided into four groups of eight animals (see table 1). After one week the rats were anaesthetized with ketamine (100 mg/kg) and xylazine (7.5 mg/kg) and underwent bilateral ovariectomy. A control group of rats underwent sham ovariectomy. One week after operation, the ovariectomized animals were subcutaneously implanted with pellets releasing either 0.25 mg β-estradiol 17-acetate (17β-estradiol) over a period of 21 days (0.01 mg/day) or 600 mg progesterone for 21 days (9.5 mg/day) (Innovative research of America, Florida, USA. The effect of placebo pellets was tested in an earlier study [15], and placebo pellets were therefore omitted in the present study. After three weeks of recovery the rats were anaesthetized with ketamine (100 mg/kg) and xylazine (7.5 mg/kg), and blood samples were taken by retro-orbital puncture. The hormone levels in the blood were analysed to ensure effect of the pellets (Progesterone EIA kit and Estradiol EIA kit (Cayman Chemical, By BioNordika Denmark A/S, Glostrup, Denmark). The measured hormone levels are shown in table 1. The anesthetized rats were sacrificed by transcardial perfusion with PBS and the uterus was rapidly taken out of the rats and flash frozen in liquid nitrogen. The tissue was pulverized in liquid nitrogen using mortar and pestle and divided into smaller portions. The pulverized tissue was stored at -−80°C until further use.

**Table 1:** Division of rats in four groups.

Group	Hormones	Plasma estradiol (pg/ml)	Plasma progesterone (ng/ml)
*Sham-operated*	No pellets implanted	81,48±125,28	55,39±45,89
*Ovariectomized (OVX)*	No pellets implanted	44,82±19,51	14,93±12,60#
*17β-estradiol-treated (E2)*	0.25 mg β-estradiol 17-acetate released in 21 days	96,32±36,78(**)	29,22±17,58
*Progesterone-treated (P4)*	600 mg progesterone released in 21 days	27,57±19,83	35,29±13,71(*)

* ^,^ **significant different from the corresponding value in OVX group.

# significant different from the corresponding value in Sham group.

Two rats from each group were used for perfusion fixation. They were treated similar to the other rats until the transcardial perfusion with PBS. After a short perfusion with PBS, these rats were fixated by perfusion with 4% paraformaldehyde in PBS. The uterus was taken out and placed in 4% paraformaldehyde in PBS for further fixation, and after a few days transferred to 70% ethanol and kept at room temperature.

### RNA Extraction

Pulverized uterus tissue was incubated for five minutes in trizol reagent. After centrifugation at 10.000×g for 10 min at 4°C, the supernatant was transferred to a Maxtract tube (Qiagen, Copenhagen, Denmark), and chloroform and RNase free water was added. Following centrifugation at 12.000×g for 10 min at room temperature the colorless water phase was transferred to a clean tube and added isopropanol. After 10 min incubation the mix was transferred to a spin column and DNase treated using SV total RNA purification kit (Promega, Nacka, Sweden). The following RNA wash was performed with SV total RNA purification kit (Promega, Nacka, Sweden) according to the guidelines from the manufacturer. Total RNA was eluted in H_2_O. Concentration and purity of the RNA was measured by spectrophotometry on a nanodrop and bio-analyzer. RIN values above 7 were accepted for further analysis. The RNA was kept at −80°C until further use.

### cDNA and qPCR

Reverse transcription of total RNA was performed using M-MLV reverse transcriptase in M-MLV RT buffer added rRNasin RNase inhibitor (Promega, Nacka, Sweden) and by use of half random hexamer and half oligodT primers (Tag Copenhagen, Copenhagen, Denmark). One µg of total RNA was added to each reaction. No-reverse transcription controls were run separately.

The cDNA was diluted ten times and amplified with Lightcycler® 480 SYBr green 1 master mix (Roche Applied Science, Hvidovre, Denmark). Specific primers were designed in Primer3 [16] and received from Eurofins (Ebersberg, Germany). The primer sequences were as follows: ACTGAGGGGTGTCAAGATGG, GTTGAGCTCCGTGATCAAGTC (SK3 forward and reverse) and ATTACTGCCCTGGCTCCTAG, CAGTGAGGCCAGGATAGAGC (β-actin forward and reverse). All primers were designed to include an exon-exon span in the amplified product. A melting curve analysis showing a single peak confirmed specificity of the primers. Standard curves for each primer set were run to ensure appropriate efficiencies. Triplicates were run for all samples and variances in C_t_ values above 0.4 were discarded. No-template control and no-reverse transcription control were run in parallel to ensure no contamination of genomic DNA. A calibrator with a mixture of cDNA from 10 rats was included in every run to take into account any day to day variation.

All data was normalized to the expression of β-actin. To test the stability of β-actin, a quantification of the initial amount of cDNA in each sample was performed using Oligreen kit (Molecular Probe, by Invitrogen, Taastrup, Denmark), as previously described [16]. β-actin qPCR data was normalised to total cDNA and found stable and suitable as reference gene. Quantification of SK3 was done by using the ratio to β-actin in each sample.

### Protein Extraction

Proteins were extracted as previously described [17], but with modifications. In short frozen pulverized tissue was homogenized in ice-cold Tris-EDTA (50 mM Tris, 1 mM EDTA in water added a complete mini protease inhibitor tablet (Roche Diagnostics, Hvidovre, Denmark) pH 7.4) with a polytron. Following centrifugation at 15.300×g for 20 min at 4°C, the supernatant was discarded and the pellet was dissolved in SDS sample buffer (125 mM Tris, 4% SDS, 20% glycerol in water added complete mini protease inhibitor tablet (Roche Diagnostics, Hvidovre, Denmark) pH 6.8). Following centrifugation at 15.300×g for 90 min at 4°C, the supernatant was collected, and protein concentration was measured using the Bradford method with BSA as standards.

### Western Blot

Proteins were separated on a 10% Bis-Tris Gel (NuPage, Invitrogen, Taastrup, Denmark) and transferred to a nitrocellulose membrane. A protein marker was run in parallel in order to determine the weight of protein bands (SeeBlue® Plus2 prestained standard, Invitrogen, Taastrup, Denmark). The membrane was blocked in 5% skimmed milk in TBS and 0.05% Tween 20 followed by incubation with the primary antibody (anti-SK3 (1∶200) Alomone Labs, Jerusalem, Israel [18] and monoclonal anti-β-actin (1∶200), Santa Cruz Laboratories by AH Diagnostics, Aarhus, Denmark) over night at 4°C and with HRP-conjugated secondary antibody (goat anti-rabbit IgG (H+L)-HRP conjugate and EIA grade affinity purified goat anti-mouse IgG (H+L)-HRP conjugate (1∶20,000), Biorad, Copenhagen, Denmark) for one hour at room temperature. The membranes were developed using Super Signal® West Pico chemiluminiscent substrate (Fisher Scientific, Slangerup, Denmark). After development, the membranes were visualized using chemiluminescence. Intensity measurements were conducted using UN-SCAN-IT gel version 6.1. All samples were normalized to the measured intensity for β-actin in the same sample.

The specificity of the SK3 antibody was tested with an absorption control using a blocking peptide (provided by Alomone Labs, Jerusalem, Israel). Antibody and blocking peptide was mixed in a 1 µg to 1 µg ratio, and was allowed to incubate over night at 4°C before used as primary antibody.

### Immunohistochemistry

The paraformaldehyde fixated tissues were dehydrated and embedded in paraffin. 5 µm thick sections were cut on a microtome and placed on Super Frost Plus™ slides. The sections were deparaffinized in a xylene/ethanol series. Antigen retrieval was performed in micro wave oven for 15 minutes in citric acid (pH 6.0). After blocking (PBS added 2% BSA) for 10 minutes, the sections were incubated with the primary antibody over night at 4°C (rabbit anti-SK3 (1∶2000) in PBS added 2% BSA, Alomone Labs, Jerusalem, Israel). After wash the sections were incubated with the secondary antibody for 40 minutes in biotinylated goat anti-rabbit IgG (H+L) (Vector Laboratories, by VWR Bie & Berntsen, Rodovre, Denmark). Following wash the sections were incubated in streptABComplex/horseradish peroxidase (Dako, Glostrup, Denmark) for 30 minutes, followed by incubation with 3.3′-Diaminobenzidine for 15 minutes. Finally the sections were counterstained with hematoxylin, dehydrated with an ethanol series and mounted with cover glass.

The specificity of the primary antibody was tested with an absorption control using a blocking peptide (provided by Alomone Labs, Jerusalem, Israel). Antibody and blocking peptide was mixed in a 1∶1 ratio, and incubated over night at 4°C by gently shaking. The antibody/blocking peptide complex were used as “primary” antibody following the staining protocol described above. To test for the signal resulting from the secondary antibody, a control was performed omitting the primary antibody.

Parallel to the immunohistochemistry staining of sections all tissue underwent staining with hematoxylin and eosin (HE) to investigate the difference in the morphology of the uterus caused by the hormones.

### Statistics

qPCR and Western Blot data was analyzed in GraphPad Prism Version 5.03 (GraphPad Software, San Diego, California, USA). Overall comparison was performed using one-way analysis of variance, and the individual groups of animals were compared with Bonferroni’s multiple comparison test. p<0.05 was considered significant.

## Results

### Histological Changes of the Uterus after Hormone Treatment

**Figure 1:**
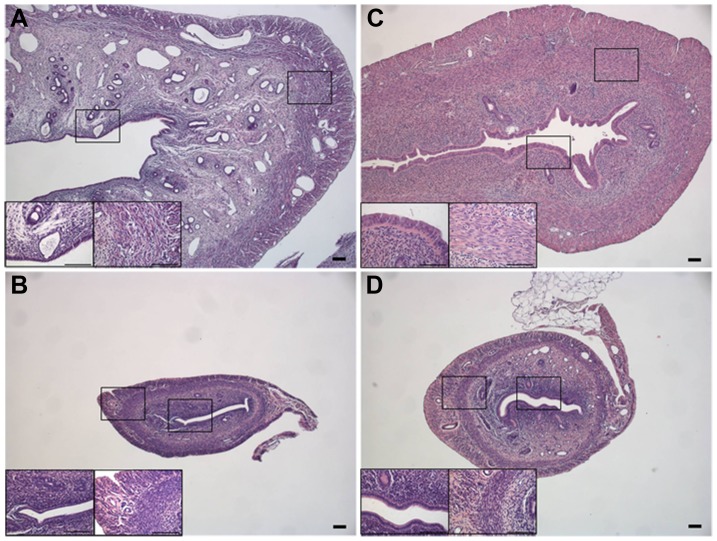
HE staining of the uterus of the four groups of rats. Sham-operated uterus showing the natural appearing uterus (A), uterus from an ovariectomized rats (B), uterus from a 17β-estradiol-treated rat (C) and D) uterus from a progesterone-treated rat (10x magnification). Inserts in A–D represent 40x magnification of epithelium and muscle layer. All scale bars represent 0.1 mm.

Tissue changes in the uterus after hormone treatment were identified with HE staining of tissue sections from each of four groups of animals (figure 1). Very clear differences in the histology between the four experimental groups were observed. The size of the whole uterus was highly influenced by estradiol, as the uterus from the sham-operated animals (figure 1A) and the animals treated with 17β-estradiol (figure 1C) was considerable larger than the uterus from ovariectomized animals (figure 1B) and animals treated with progesterone (figure 1D). Furthermore, the tissue from the ovariectomized rats and rats treated with progesterone seemed more condensed with smaller cell sizes than the uteri of the sham operated animals and those treated with 17β-estradiol. This was apparent both in the myometrium and in the epithelium of the uterus (see insets for all groups in figure 1). Numbers of nuclei in the epithelium and circular smooth muscle layer, nuclei sizes and size of muscle layer was estimated from the HE staining’s and are presented in table 2.

**Table 2:** Estimated changes in tissue morphology after hormonal treatment.

Group	Thickness of circular smooth muscle tissue	Number of nuclei per measureddistance in circular muscle layer	Number of nuclei per measureddistance in luminal epithelium	Size of nuclei in luminal epithelium
*Sham*	200 µm	1	1.3	5–6 µm
*OVX*	90 µm	3.5	1.8	3–4 µm
*E2*	175 µm	0.5	1	9–10 µm
*P4*	80 µm	2.2	3.1	5–6 µm

### Down-regulation of SK3 mRNA in Rats Treated with 17β-estradiol

**Figure 2:**
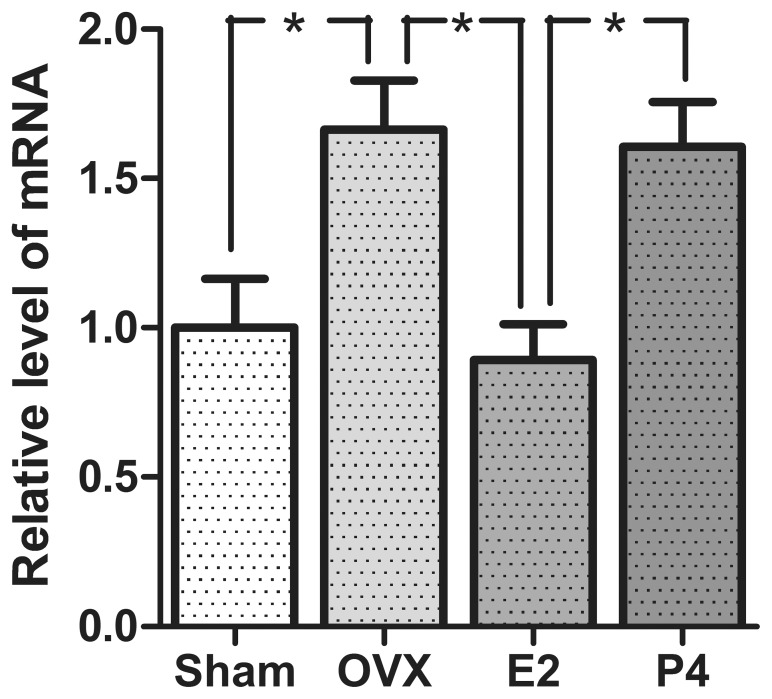
mRNA levels of SK3 in the rat uterus of the different groups of rats. All data are ratio to β-actin in the same sample and sham values are set to 1. Data are presented as mean ± S.E.M. Asterisk (*) represent significant difference in the different groups of animals (p<0.05). n = 6, 5, 6 and 6 for sham-operated animals, ovariectomized animals (OVX), 17β-estradiol-treated animals (E2) and progesterone-treated animals (P4) respectively.

To examine the hormonal effect on the mRNA level of SK3 in the uterus qPCR was conducted. The obtained results are shown in figure 2. The mRNA level for SK3 was greatly influenced by 17β-estradiol, as the mRNA level in the 17β-estradiol-treated animals was significantly lower than the level in the ovariectomized rats. Additionally a significant difference in mRNA level was observed between the sham-operated rats and the ovariectomized rats. No difference between the ovariectomized animals and the progesterone-treated animals was observed, indicating that progesterone does not affect the mRNA level of SK3.

### Up-regulation of SK3 Protein in Rats Treated with 17β-estradiol

**Figure 3:**
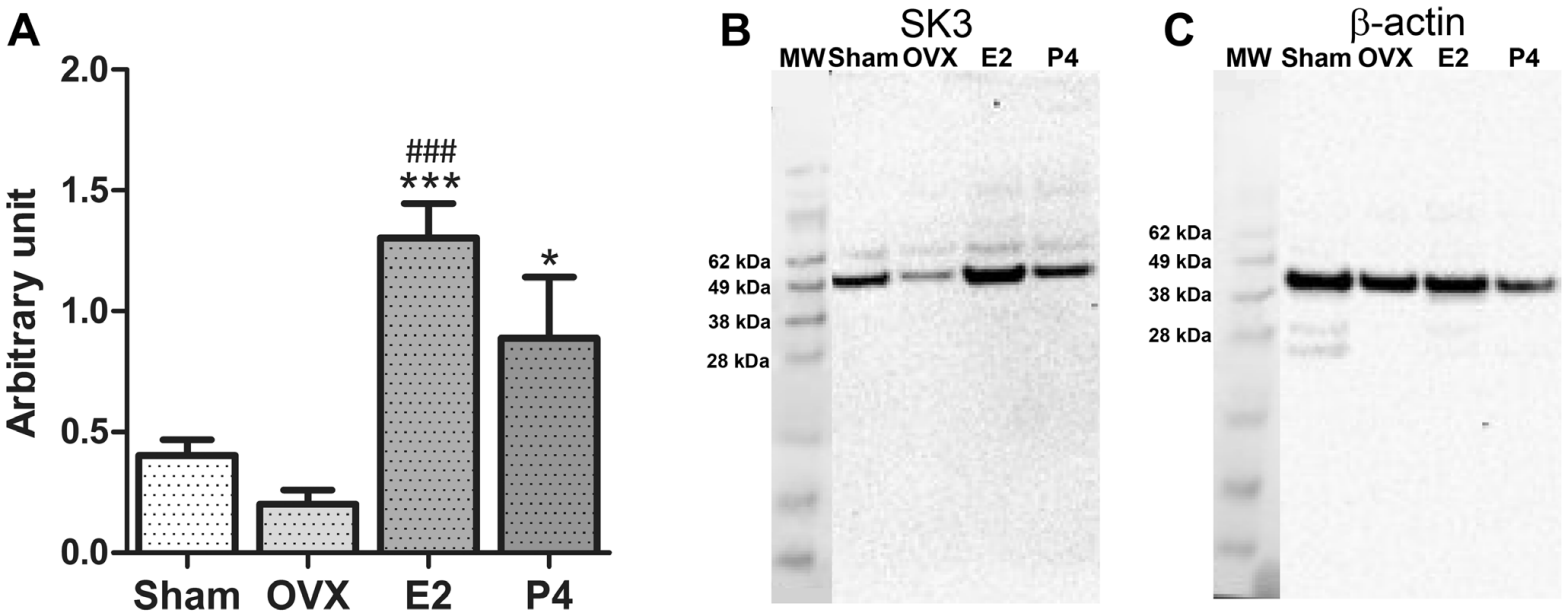
SK3 protein expression in the four groups of animals. (A) Accumulated measurements of protein expression from all groups. All data are ratio to β-actin in the same sample. Data are presented as mean ± S.E.M. Asterisk (*) represent significant difference from the oveariectomized rats (*p<0.05, ***p<0.001). Number sign (#) represent significant difference from the sham-operated rats (### p<0.001). n = 6, 4, 6 and 3 for sham-operated animals, ovariectomized animals (OVX), 17β-estradiol-treated animals (E2) and progesterone-treated animals (P4) respectively. (B, C) Representative western blot showing the protein bands for SK3 (B) and β-actin (C) in the four groups of rats. (Protein marker was used to determine molecular weight (MW) of the protein).

To investigate hormonal influence on the SK3 protein western blot was performed. A significantly higher amount of SK3 protein expression was observed in the animals treated with 17β-estradiol compared to the protein amount observed in the sham-operated animals and the ovariectomized animals (figure 3). No significant difference in protein expression was observed between the sham-operated animals and the ovariectomized animals. Surprisingly the SK3 protein expression in the progesterone treated animals was also significantly higher than the level in the ovariectomized animals.

The intensity measurements of β-actin bands showed no significant difference between the four groups of rats (data not shown).

### SK3 is Localized in the Endometrial Epithelium

**Figure 4:**
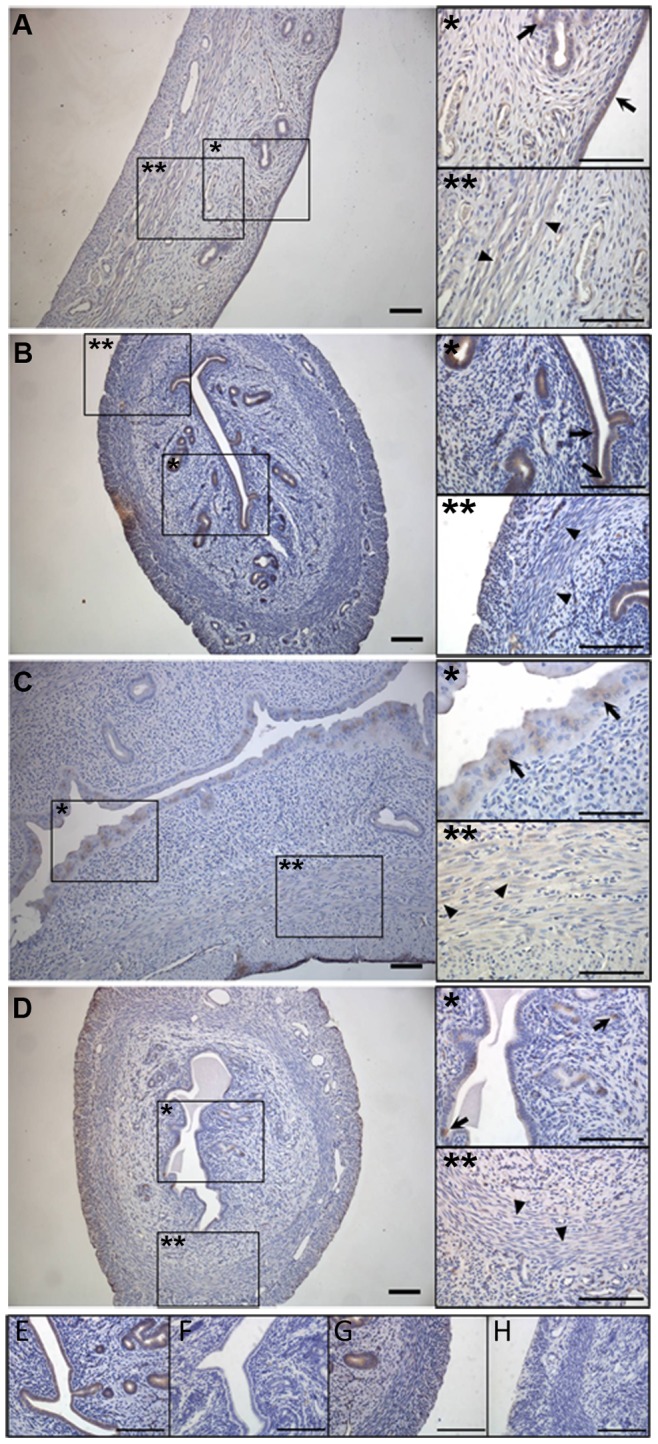
Immuno staining of rat uterus in the four groups of animals showing localization of SK3. SK3 staining of sham-operated animals (A), of ovariectomized animals (B), of 17β-estradiol-treated animals (C) and of progesterone-treated animals (D). A–D is shown in 10x magnification. Inserts show 40x magnification of muscle layer (**) and epithelium (*). E–H) Absorptions control using specific blocking peptide. Positive SK3 staining in a 1∶8000 dilution showing signal in both epithelium and smooth muscle layer (E and G). Peptide absorptions control, a 1∶8000 dilution of antibody/control peptide complex eliminates the staining in epithelium and muscle (F and H). Controls are shown in 40x magnification. All scale bars represent 0.1 mm. Arrows point at epithelial localization, while arrow heads point at muscular localization.

An examination of the localization of SK3 in the rat uterus was conducted using immunohistochemistry. Specific staining for SK3 was observed in both the luminal and glandular epithelium in all four groups of animals (figure 4, inserts). Furthermore a weak staining was observed in the smooth muscle layer in all the four groups of animals. Though hard to distinguish from background staining, this signal was not present in the preabsorption control using immunogene peptide, which indicates specific staining (see preabsorption control in Figure S1).

The specificity of the antibody against SK3 was evaluated by a liquid phase preabsorption control using the immunogene peptide (see figure 4E–H). The controls were made with a final dilution of antibody/immunogene peptide complex at 1∶8000, whereas all samples were made in a 1∶2000 dilution. The stained structures in the sections were identical in the two dilutions however the signal was stronger with the 1∶2000 dilutions. So for visual reasons a 1∶2000 dilution was chosen for the samples.

## Discussion

In the present study we have investigated the presence and localization of SK3 in the uterus of rats stimulated with 17β-Estradiol and progesterone. We found a down-regulation of SK3 mRNA in the animals treated with 17β-estradiol, whereas no effect was observed in the animals treated with progesterone compared to the ovariectomized animals. Strikingly, at the same time we found an increase of SK3 protein after stimulation with 17β-estradiol as shown with western blot. SK3 were localized to the endometrial epithelium in all the four groups of rats. In addition in all the groups we detected a weak signal in the myometrium.

To ensure correct release of hormones from the pellets, blood samples were obtained from the rat just prior to the sacrificing. Blood analysis from ovariectomized rats showed a significantly lower level of progesterone than the corresponding values in the sham-operated animals. This was not observed for the estrogen values, probably due to the high variances in plasma estrogen level seen in the sham-operated animals as a result of the sham animals being in different phases of estrous. Blood analysis from animals treated with 17β-estradiol and progesterone revealed hormonal plasma values that were significantly higher than the values from the ovariectomized animals. Yet, as the hormone values were somewhat lower than expected from the amount of hormones implanted in the animals, HE staining were done to visualize the effect of hormones on the uterus tissue. A clear effect of the hormonal treatment was observed on the morphology of the tissue. The uterus from the ovariectomized animals and the animals treated with progesterone appeared smaller and denser, which could indicate that a degree of atrophy had taken place in the tissue of these animals. Furthermore this was clear from the number of nuclei in the epithelium and the circular smooth muscle layer (table 2). In contrast, the uterus from the 17β-estradiol-treated animals resembled that of the sham animals in the size of circular smooth muscle layer and the number of nuclei. However, in the epithelium a lower number of nuclei were estimated at a measured distance and an increase in size of the nuclei was apparent. This indicates that some hypertrophy had taken place in the 17β-estradiol-treated tissue. These changes in the tissue correspond to the measured changes in tissue from hormone stimulated rats reported by Vedernikov *et al.* [19], which support the effect of the hormones.

Studies in mice and human myometrium have reported a down-regulation of SK3 mRNA and protein expression during pregnancy [9], [13], [20]. However, the single study made in rats reported no change in SK3 expression during pregnancy neither at mRNA level nor protein level [12]. In the present study we found an up-regulation of SK3 protein expression in the animals stimulated with 17β-estradiol. Surprisingly this up-regulation of SK3 was not accompanied by a corresponding increase in mRNA level. In fact the amount of SK3 mRNA decreased after stimulation with 17β-estradiol. A previous study show that mRNA and protein levels for SK3 do not always correspond [21]. In addition, though previous studies show a down-regulation of SK3 mRNA and protein following 17β-estradiol stimulation in the myometrium, other reports demonstrate an up-regulation [22], [23], supporting our findings and show that the influence of 17β-estradiol on SK3 expression is rather complex.

The 17β-estradiol released in the animals might cause multiple alterations in the cell, which may help to explain the contradictory findings for SK3 on protein level and mRNA level.

Recently there has been a lot of focus on the microRNAs and their role in regulation of transcription and translation. Even though microRNAs are traditionally known as posttranscriptional regulators [24], research have shown that some are able to augment global protein synthesis by the binding to ribosomal protein mRNA and enhance their translation [25]. The expression of several microRNAs in the uterus is under the influence of estrogen [26]. Thus, the expression of SK3 protein in the uterus may be held high in the 17β-estradiol-treated animals because of an increase in the translation of ribosomal protein, caused by microRNA. Another possible explanation is a decrease in the SK3 turnover as a result of the treatment with the 17β-estradiol.

No change in SK3 mRNA was detected for the progesterone-treated animals, however an increase was observed at the protein level. To the authors’ knowledge, the regulation of SK channels by progesterone has only been scarcely investigated. In a functional study in human myometrium from pregnant women, apamin among other ion channel inhibitors failed to counteract the inhibitory effect of progesterone on contractions [14]. From this it was suggested that progesterone did not work through SK channels or a number of other K^+^ channels. Despite this finding, from our study it appears that progesterone has a stimulating effect of SK3 protein expression in rat uterus.

Using immunohistochemistry we localized SK3 in the glandular and luminal epithelium of the endometrium. This location of the SK3 channels in the uterus has only been mentioned in a few previous studies. Palmer *et al*. identified SK3 channels in immortalized porcine endometrial gland epithelial monolayers [28], and recently Noble *et al.* [12] found a weak staining of SK3 in the glandular epithelium in rats, indicating the presence of the channels here. However, this was not discussed further in their study.

Interestingly the epithelial localization was found in all the four treated groups of animals. Some difference in the staining arrangement was seen among the different rats. However, as observed in figure 1 and table 2, the hormone treatment resulted in very obvious changes in the tissue both in density of the nuclei, and the nuclei sizes. These differences are likely to be the reason for the variances in staining pattern. No apparent difference in staining intensity was observed among the groups however in order to elucidate potential changes in the expression of SK3 in the endometrial epithelium quantitative techniques like e.g. autoradiography should be performed.

In addition to the epithelial staining, we observed a weak signal in the myometrium. Though this signal was difficult to distinguish from background staining, it disappeared when performing the peptide control, which urged us to believe, that this staining is specific. Furthermore, a potential localization of SK3 in the smooth muscles corresponds to earlier studies in rats and mice where specific SK3 staining was identified within the myometrium [10], [12]. In addition, Skarra *et al.* [27] localized SK3 to the plasma membrane of the smooth muscle cell of the myometrium in mice. Interestingly a recent study in humans localize the SK3 in the myometrium to the telocytes and propose that SK3 is absent from the muscle cells [13]. In our study, we did not observe telocyte localization, but we cannot exclude the possibility of the channels being present in these cells.

The present identification of SK3 expression in the endometrial epithelium together with the possible role in the myometrium suggests multiple functions of the channel in the uterus.

In addition to contributing to the quiescence of the myometrium, we suggest a role for SK3 in the endometrial epithelium possibly in K^+^ secretion or recycling. Palmer *et al.* identified apical SK3 in immortalized porcine endometrial gland epithelial cells, where they were suggested to contribute to K^+^ secretion to the uterine fluid [28]. Furthermore, several K^+^ channels have been identified in epithelial cells in organs such as the kidney, where they mediate K^+^ recycling and secretion [29]. Thus expression of SK3 in the endometrial epithelium makes it plausible to picture a similar role of SK3 in the uterus.

Recently we have identified SK3 in the glandular epithelium of human non-pregnant endometrium [30], and the finding of the same pattern in the rat endometrium makes the rat a potential model for studies in the endometrial epithelium, also in humans. Furthermore, it strengthens our present findings, and the suggested roles of SK3 in this tissue. Lastly, the presence of the channels in the epithelium of both species could point to the possibility that SK3 contribute to the contractions of the myometrium in an indirect manner by possible secretion and uptake from the uterine lumen. This suggestion is reinforced by our findings in the human myometrium, where no difference was observed in the level of SK3 mRNA between tissue from pregnant women at term laboring and at term non-labouring [30].

In the present study we did not find the same expression pattern of SK3 as in the previous study in rat uterus [12]. In the previous study natural pregnant rats were used, whereas the rats used in our study were virgin rats stimulated with 17β-estradiol or progesterone. From our study we can conclude, that the natural pregnant rats are more complex, and that they have other regulatory mechanisms than the ones we can simulate by addition of hormones. Despite of this, the hormone-stimulated animals are useful tools to learn more about the nature and regulation of the proteins.

In summary we have localized SK3 to the endometrial epithelium of the rat uterus. Furthermore we have identified the channels in the glandular and luminal endometrial epithelium. We found opposing regulation of SK3, with a down-regulation at transcript level and up-regulation at protein level. In addition to the contribution to uterine quiescence reported by others, we suggest a role for the epithelial SK3 channels in K^+^ recycling and/or K^+^ secretion.

## Supporting Information

Figure S1 Absorption control on epithelium and muscle tissue. Positive SK3 staining in a 1:8000 dilution in epithelium (A) and smooth muscle (B). Peptide absorption control, in 1:8000 dilution in epithelium (C) and smooth muscle (D). Illustrations are shown in 40x magnification. All scale bars represent 0.1 mm.
(TIF)
