# Supplementary figures and images for: Expression of the Small Conductance Ca2+-Activated Potassium Channel Subtype 3 (SK3) in Rat Uterus after Stimulation with 17β-Estradiol
Source: PLoS One. 2014 Feb 5;9(2):e87652. doi: 10.1371/journal.pone.0087652 (PMC3914860; doi:10.1371/journal.pone.0087652)

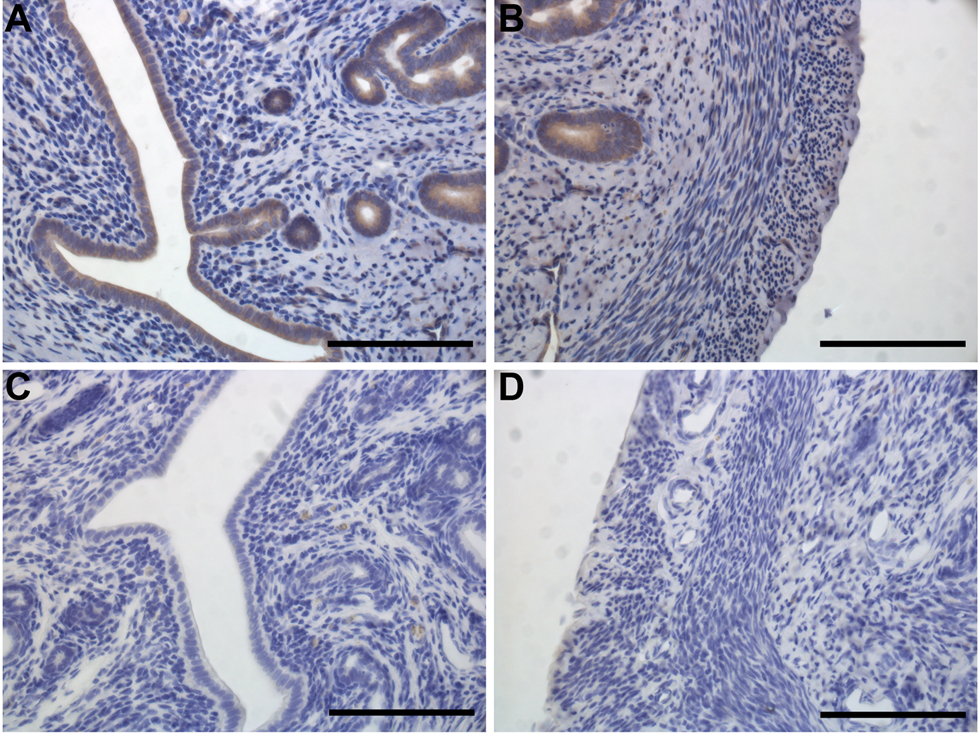

Supplement: Figure S1 — Absorption control on epithelium and muscle tissue. Positive SK3 staining in a 1:8000 dilution in epithelium (A) and smooth muscle (B). Peptide absorption control, in 1:8000 dilution in epithelium (C) and smooth muscle (D). Illustrations are shown in 40x magnification. All scale bars represent 0.1 mm. (TIF) [file pone.0087652.s001.tif]
